# The Difference between the Population Attributable Risk (PAR) and the Potentioal Impact Fraction (PIF)

**DOI:** 10.18502/ijph.v49i10.4713

**Published:** 2020-10

**Authors:** Maryam ASKARI, Seyedeh Mahdieh NAMAYANDEH

**Affiliations:** Yazd Cardiovascular Research Center, Afshar Hospital, Shahid Sadoughi University of Medical sciences, Yazd, Iran

## Dear Editor-in-Chief

Two types of measure of association are used to estimate the effect of exposure in the occurrence of the disease. 1) Relative difference or incidence ratio and 2) absolute difference of the incidence of disease. Relative differences or ratios indicate the strength of association, and are present in etiological studies. absolute differences, bring a perspective of population prevention strategies (1).

Attributable Risk (AR) based on absolute differences. AR it should be interpreted as a true etiologic fraction. AR in Exposed Individuals (AR_exp_) indicates the number of cases of a disease among exposed individuals that can be attributed to that exposure. Population Attributable Risk (PAR) is the porportion of the incidence of a disease in the population (exposed and nonexposed) that is due to exposure. PAR is the difference between the risk in the total population and that in unexposed subjects. It is the incidence of a disease in the population that would be eliminated if exposure were eliminated (1, 2).

Equation 1: PAR =Incidence rate in total population−Incidence rate unexposed

The population Attributable Fraction (PAF) is the proportion of cases for an outcome that can be attributed to a certain risk factor among the entire population (3).

Equation 2: $$\mathrm{PAF}=\frac{\mathrm{Incidence}\;\mathrm{rate}\;\mathrm{in}\;\mathrm{total}\;\mathrm{population}\;-\;\mathrm{Incidence}\;\mathrm{rate}\;\mathrm{unexposed}}{\mathrm{Incidence}\;\mathrm{rate}\;\mathrm{in}\;\mathrm{total}\;\mathrm{population}}$$

Equation 3: $$\mathrm{PAF}=\frac{P\left(RR-1\right)}{P\left(RR-1\right)+1}$$

If there was many exposure the following formula applies:

Equation 4: $$\mathrm{PAF}=\frac{\sum_{i=1}^{n}P_{i}\left(RR-1\right)}{\sum_{i=1}^{n}P_{i}RR_{i}}$$

The potential impact fraction (PIF) is another measure that is calculated based on both the prevalence of a risk factor and the associated relative risk. The PAF is a special case of the PIF. PIF Is a reduction in the fraction of a disease that results from a proportional change in disease incidence when exposure to a risk factor is changed (4).

And its equation is as follows:

Equation 5: $$\mathrm{PIF}=\frac{\left(P-P^{\prime }\right)\left(RR-1\right)}{P\left(RR-1\right)+1}$$

P is the prevalence of the risk factor and P′ the counterfactual, RR is the relative risk.

So, there are many interpretations for each of these concepts. PAF provides important information about the potential impact of prevention programs and interventions in public health, being extremely useful for policymakers, managers and decision makers.

Therefore, it would be possible to estimate PAF using epidemiological data on the prevalence of exposure and the relative risk of disease.

## References

[1] Kaelin MA Bayona M (2004). Attributable risk applications in epidemiology. College Entrance Examination Board. 6– 7.

[2] Szklo M Nieto FJ Miller D. Epidemiology: beyond the basics. Oxford University Press; 2001.

[3] Porta M. A dictionary of epidemiology: Oxford university press; 2008 Pp. 12– 3.

[4] Veerman JL Barendregt JJ Van Beeck EF Seidell JC Mackenbach JP (2007). Stemming the obesity epidemic: a tantalizing prospect. Obesity, 15( 9): 2365–70. 17890506 10.1038/oby.2007.280

